# Mutual Information and Multi-Agent Systems

**DOI:** 10.3390/e24121719

**Published:** 2022-11-24

**Authors:** Ira S. Moskowitz, Pi Rogers, Stephen Russell

**Affiliations:** 1 Naval Research Laboratory, Code 5580, Washington, DC 20375, USA; 2 2022 SEAP Summer Intern at the Naval Research Laboratory, Washington, DC 20375, USA; 3 Jackson Health System, 1500 NW, 12th Ave, Miami, FL 33136, USA

**Keywords:** multi-agent system, mutual information, channel capacity, information geometry

## Abstract

We consider the use of Shannon information theory, and its various entropic terms to aid in reaching optimal decisions that should be made in a multi-agent/Team scenario. The methods that we use are to model how various agents interact, including power allocation. Our metric for agents passing information are classical Shannon channel capacity. Our results are the mathematical theorems showing how combining agents influences the channel capacity.

## 1. Introduction

Advances in machine intelligence have led to an increase in human-agent teaming. In this context, one or more machines act as semi-autonomous or autonomous agents interacting with other machine teammates and/or their human proxies. This phenomenon has led to cooperative work models where the role of an agent can be, interchangeably, a human, or machine, support system. Human counterparts that interact with automation become less like operators, supervisors, or monitors, and more like equal-authority peers.

Critical to the success of any team is efficient and effective communication. Multi-agent systems are no different. Information sharing is a key element in building collective cognition, and it enables agents to cooperate and ultimately achieve shared goals successfully. Information sharing, or communication, provides the foundation for a team’s success. In complex multi-agent engagements, information is not always universally available to all agents. Such engagements are often characterized by distributed entities with limited communication channels among them, where no agent has a complete view of the solution space, and information relevant to team goals only becomes available to team members in spontaneous, unpredictable and even unanticipated ways. Moreover, there is always a resource cost to inter-agent communication. Finding highly efficient and effective communication patterns is a recurring problem in any multi-agent system, particularly if the system agents are distributed.

We are concerned with how a Multi-agent System (MAS) [1], or Team, sends information between agents or teammates. By “how” we mean “how” in an information theoretic [2] sense—in particular, we do not concentrate on the mechanics or physics of the transmission other than how it impacts information theory. We are concerned with what strategy an agent can to use to maximize its information flow to another agent. From an information geometric standpoint, we only use a simple metric in this article, but lay the ground work for more complex Riemannian metrics. We are concerned with a transmitting agent sending a small amount of distinct symbols in a fixed time. In fact, we restrict ourselves to two symbols to develop our theory (A list of notation is at the end of the article.). We are using a mathematical approach to model the communication between two agents. The equations we present are based on a series of assumptions that we will explain.

We assume that an agent sends two symbols to another agent. We refer to the symbols as “0” or “1”. We are concerned with the fidelity of how the symbols are passed. All symbols take the same time to pass. We will be looking at the (Shannon) capacity as one agent attempts to send a symbol to another agent.

**Figure 1:**
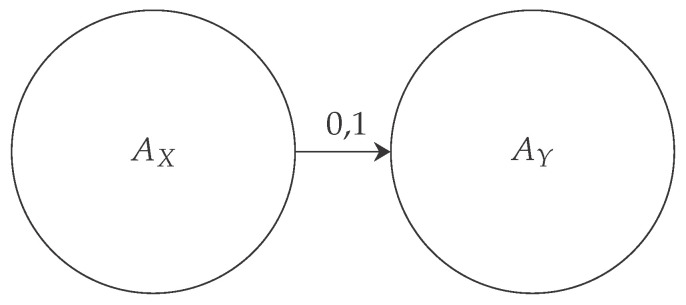
Heuristic figure of $A_{X}$ transmitting a bit to $A_{Y}$.

**Figure 2:**
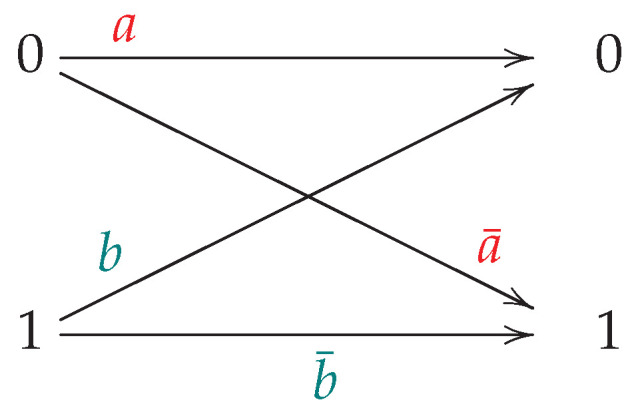
The noisy channel diagram corresponding to the first figure.

Our scenario is illustrated in Figure 1 and Figure 2. The first agent $A_{X}$ sends a 0 or 1 to the second agent $A_{Y}$. We have a clock and the unit of time is *t*. Every *t*, $A_{X}$ transmits the symbol to $A_{Y}$. We assume that the symbol is received within the same time unit (i.e., we assume instantaneous transmission speeds during each interval *t*). There is no feedback (which, for the channels we analyze, would not change the capacity anyway (p. 520 [3])) from $A_{Y}$ to $A_{X}$, and the transmission is considered to be memoryless (quoting [4], “…channel is memoryless if the probability distribution of the output depends only on the input at that time and is conditionally independent of previous channel inputs or outputs”). Furthermore, it is implicit that the channel statistics never change (sometime the literature refers to this as a “stationary” condition).

To summarize the above, we have a Discrete Memoryless Channel (DMC) between $A_{X}$ and $A_{Y}$. This channel measures information flow in terms of bits per symbol (since *t* does not vary). We let *X* represent the input distribution to this DMC, and we let *Y* denote the output random variable.

The probability for the random variable *X* is given by P(X=i),i=0,1; it is the probability that $A_{X}$ inputs symbol *i*, and P(Y=j),j=0,1 is the probability that $A_{Y}$ received symbol *j*. The input distribution *X* is determined by the transmission fidelity of $A_{X}$. In particular,

(1) $$x=P\left(X=0\right)=x,\;\bar{x}:=P\left(X=1\right)=1-x\;.$$

Whereas the output distribution *Y* is determined by the (assumed to be well-defined) conditional distribution between *X* and *Y*, and the input distribution. Thus,

(2) $$P\left(Y=j\right)=\sum_{i}P\left(Y=j|X=i\right)\cdot P\left(X=i\right)\;.$$

The approach presented in this paper follows from [2,5,6,7].

The conditional probabilities of the DMC is given by a 2×2 matrix $M_{1}$, where (Please keep in mind the swapping of the indices, and, as we had above for $\bar{x}$, that notationally $\bar{*}:=1-*$. Furthermore, the convention is that a conditional probability is fixed for all P(X=i), even if that probability is 0. In the next footnote, we address the impact of this with respect to (w.r.t.) information theory).

(3) $$m_{i,j}:=P\left(Y=j|X=i\right)\;\mathrm{and}\;$$

(4) $$M_{1}=\left(\begin{array}{cc} m_{0,0} & m_{0,1}\\ m_{1,0} & m_{1,1} \end{array}\right)=\left(\begin{array}{cc} P(Y=0|X=0) & P(Y=1|X=0)\\ P(Y=0|X=1) & P(Y=1|X=1) \end{array}\right)=:\left(\begin{array}{cc} a & \bar{a}\\ b & \bar{b} \end{array}\right)\;.$$

Note that (a,b)∈[0,1]×[0,1].

Before we continue with the mathematics let us put this research into some more perspective. Von Neumann’s [8] seminal work had no concept of “Teamwork”, which is at the core of what we are discussing. Sliwa’s [9] review suggests that minimum communication channels are more important when context is understood during teamwork, a suggestion opposite to our work in this article which we hope to test in the future. Lawless [10] suggests that maximized channels become more important when Teams confront uncertainty in their environment. Schölkopf et al. [11] suggest that i.i.d. data are insufficient to reconstruct whatever social event is being captured, that something is missing and a new approach must be innovated, our goal in this article. Our results will be discussed in situ for maximum effect.

### 1.1. Entropy and Mutual Information

We extend our random variables to allow more than two possible outcomes, and give the following definitions with the most generality possible. We now have I+1 possible inputs, and J+1 possible outcomes.

Given a discrete random variable *V*, we define the entropy of *V* as (By convention log is the base 2 logarithm, and ln is the natural logarithm. Furthermore, we are able to extend the definitions (p. 19 [4]), as is standard, so that 0log(0)=0log(0/0)=0. These conventions allows the most general derivation of (8) from (7)).

$$H\left(V\right):=-\sum_{j}P\left(V=v_{j}\right)\log P\left(V=v_{j}\right)\;.\;$$

If z∈[0,1], then we define the binary entropy function of *z* as

h(z):=−zlog(z)−(1−z)log(1−z).

Note that if *B* is a binary random variable taking the values 0 or 1, then $H\left(B\right)=h\left(P(B=0)\right)$. In fact, we simplify the notation and express the probability of the event $\left\{V=v_{k}\right\}$ as

$$p_{v}\left(v_{k}\right)=P\left(V=v_{k}\right)\;.$$

Furthermore, when it is clear which distribution we are using, we further simplify the notation and just write $p(v_{k})$. Thus,

$$H\left(B\right)=h\left(p(0)\right)\;.$$

Given two discrete random variables V,W, we define [2] the conditional entropy of *V* given *W* as

(5) $$\begin{array}{cc} H(V|W):= & -\sum_{i}p_{w}\left(w_{i}\right)\sum_{j}p_{v|w}\left(v_{j}|w_{i}\right)\log p_{v|w}\left(v_{j}|w_{i}\right)\;, \end{array}$$

where, as in the 2×2 case

$$P\left(v_{j}|w_{i}\right):=m_{i,j},i=0,1,\ldots ,I;j=0,1,\ldots ,J,$$

forming the channel matrix (Of course, as in the 2 × 2 case, conditional probability is only defined when $p(w_{i})\neq 0$. However, as we note below, such terms are dealt with by using the limiting value of the constant conditional probability term which makes our mutual information calculations consistent, keeping in mind that 0log∗ is always taken to be 0. Furthermore, keep in mind that a distribution that achieves capacity for a 2-input channel (the subject of this paper) never has either probability value as zero of course (Ref. [12] gives better bounds). There are, however, 3×2 channels for which this does not hold, for example $\left(\begin{array}{cc} 1 & 0\\ 0.8 & 0.2\\ 0 & 1 \end{array}\right)$ which has an optimizing input distribution of (0.5,0,0.5).)

(6) $$M=\left(\begin{array}{cccc} p(v_{0}|w_{0}) & p(v_{1}|w_{0}) & \ldots & p(v_{J}|w_{0})\\ p(v_{0}|w_{1}) & p(v_{1}|w_{1}) & \ldots & p(v_{J}|w_{1})\\ \vdots & \vdots & \ddots & \vdots \\ p(v_{0}|w_{I}) & p(v_{1}|w_{I}) & \ldots & p(v_{J}|w_{I}) \end{array}\right)\;.$$

We define the mutual information between *V* and *W* by [2]

(7) I(V,W):=H(V)−H(V|W)=H(W)−H(W|V)=:I(W,V).

Using (5) and (7), and some substitutions [4] (again, division by 0 is taken care of in the usual way by using limiting values ([Section 2.3] [4]), we find that

(8) $$I\left(V,W\right)=\sum_{j,i}p\left(v_{j},w_{i}\right)\log\left(\frac{p(v_{j},w_{i})}{p\left(v_{j}\right)p\left(w_{i}\right)}\right)\;.$$

We now give Shannon’s definition [2] of (channel) capacity. It has been well-studied since its inception. We will not delve into the Noisy Coding Theorem, or any of the other results which showcase its importance. Rather, we will assume in this paper that capacity is a standard measure of how much information a channel can transmit in an essentially noise-free manner [2,4]. The traditional units of capacity and mutual information are accepted in this article; they are bits per channel usage, which in our scenario is equivalent to bits per *t*.

**Definition** **1**. *We consider W to be the input random variable to a DMC. The capacity C of the DMC is*

(9) $$C:=\underset{\left\{p\left(w_{i}\right)\right\}}{\sup}I\left(V,W\right)\;.$$

The optimization is taken over all possible distributions of *W* with its fixed values $w_{i}$. The supremum is actually achieved and can be taken as a maximum [2,4]. Note that when trying to compare the magnitude of the channel capacity (with the same number of inputs), it suffices to compare the mutual information for all *x* values. Of course the two channels may have different optimizing distributions. Note the principle (and similar principles) that if $\forall x,I\left(CH_{1},x\right)\leq I\left(CH_{2},x\right)$ and if $CH_{1}$ achieves capacity at $x^{\prime }$, then $C\left(CH_{1}\right)=I\left(CH_{1},x^{\prime }\right)\leq I\left(CH_{2},x^{\prime }\right)\leq C\left(CH_{2}\right)$.

Of course swapping rows, or swapping columns from the channel matrix (6) is just notational and leaves capacity unchanged. However, we end this subsection with some interesting results in information theory—some obvious, some not so obvious.

**Property** **1**. *Removing a row from the channel matrix (6) never increases the capacity.*

**Proof.**  Not using a channel input cannot increase mutual information. This is equivalent to using input probability distributions which are always zero for a particular index; therefore, the capacity can never be greater since capacity is the maximum over all input distributions. □

**Property** **2**.

*A*— *For any input probability, combining (by adding two columns to form one column hence reducing the channel matrix from n×m to n×m−1 as illustrated below with $Q,Q^{\prime }$) two columns of a channel matrix will never increase mutual information.*
*B*— *For input probabilities with all terms non-zero, the mutual information will stay the same iff one of the combined columns is a multiple of the other. Otherwise, the uncombined channel has a larger mutual information and hence a larger capacity. (Note, that for a 2-input channel [12] has shown that the capacity achieving distribution has both probabilities in the interval $\left[\frac{1}{e},1-\frac{1}{e}\right]$ so we can apply this property to the capacity directly.)*

**Proof.**  A:

$$\left(\begin{array}{ccc} a & b & c\\ d & e & f\\ g & h & i \end{array}\right)\cdot \left(\begin{array}{cc} 1 & 0\\ 1 & 0\\ 0 & 1 \end{array}\right)=\left(\begin{array}{cc} a+b & c\\ d+e & f\\ g+h & i \end{array}\right)$$

The Data-Processing Inequality (Cascade of Channels) [3] shows that the capacity of the third channel above cannot be greater than that of the first channel. That is, processing one channel into another can never increase the information sent. The actual statement of the inequality is for mutual information. However, we use the probability that maximizes the mutual information of the first channel (which is its capacity), and therefore, it is less than or equal to the mutual information of the third channel which is less than or equal to the third channel’s capacity. This argument holds for any initial channel matrix (with adjustments to the second matrix), not just the 3×3 matrix, or the columns we chose, for simplicity above.

B: Without loss of generality (WLOG), combine the first two columns of *n* by *m* channel matrix (note how the indices are reversed as compared to (6))

$$Q=\left(\begin{array}{cccc} q_{11} & q_{12} & \cdots & q_{1m}\\ q_{21} & q_{22} & \cdots & q_{2m}\\ \vdots & \vdots & \ddots & \vdots \\ q_{n1} & q_{n2} & \cdots & q_{nm} \end{array}\right)\;\left(\mathrm{uncombined}\right)\;$$

to make

$$Q^{\prime }=\left(\begin{array}{ccc} q_{11}+q_{12} & \cdots & q_{1m}\\ q_{21}+q_{22} & \cdots & q_{2m}\\ \vdots & \ddots & \vdots \\ q_{n1}+q_{n2} & \cdots & q_{nm} \end{array}\right)\;\left(\mathrm{combined}\right)\;.$$

For *Q*, the output symbols are $y_{j}$, where *j* goes from 1 to *m*. For $Q^{\prime }$, they are the same, but with $y_{1}$ and $y_{2}$ replaced by $y_{1}\cup y_{2}$. For both channels, the input symbols are $x_{i}$, with input probability vector *p* defined as

$$p_{i}:=p\left(x_{i}\right).$$

Therefore,

$$p\left(y_{1}\right)=\sum_{i=1}^{n}p_{i}q_{i1}$$

and

$$p\left(y_{2}\right)=\sum_{i=1}^{n}p_{i}q_{i2}\;.$$

If either of these last two relations are 0, WLOG we assume $p(y_{1})=0$. This assumption means column 1 of *Q* must be a 0 column (since the input probabilities are positive), so it contributes 0 to the mutual information. Therefore, the mutual informations are equal, and one column is a constant multiple of the other. Now that we have dealt with this case, we can assume $y_{1}$ and $y_{2}$ are positive for the remainder of this proof. For fixed *p*, the mutual information of an *n* by *m* channel is

$$I=\sum_{i=1}^{n}\sum_{j=1}^{m}p\left(x_{i}\right)p\left(y_{j}|x_{i}\right)\log\frac{p(y_{j}|x_{i})}{p(y_{j})}\;.$$

Columns 3 through *m* of *Q* and 2 through m−1 of $Q^{\prime }$ are the same, so their contributions to mutual information are the same. Therefore, we only need to consider columns 1 and 2 of *Q* and column 1 of $Q^{\prime }$. Let $\bar{I}$ be these columns’ mutual information, that is,

$$\bar{I}\left(Q\right)=\sum_{i=1}^{n}p_{i}q_{i1}\log\frac{q_{i1}}{p(y_{1})}+\sum_{i=1}^{n}p_{i}q_{i2}\log\frac{q_{i2}}{p(y_{2})}\;,\;\mathrm{and}\;$$

$$\bar{I}\left(Q^{\prime }\right)=\sum_{i=1}^{n}p_{i}\left[\left(q_{i1}+q_{i2}\right)\log\frac{q_{i1}+q_{i2}}{p\left(y_{1}\right)+p\left(y_{2}\right)}\right]\;,\;\sin\mathrm{ce}\;p\left(y_{1}\cup y_{2}\right)=p\left(y_{1}\right)+p\left(y_{2}\right),\mathrm{etc}.$$

Note that $\bar{I}\left(Q\right)$ can also be written as

$$\bar{I}\left(Q\right)=\sum_{i=1}^{n}p_{i}\left[q_{i1}\log\frac{q_{i1}}{p(y_{1})}+q_{i2}\log\frac{q_{i2}}{p(y_{2})}\right].$$

The log sum inequality [4] states that, for a series of non-negative numbers $a_{k}$ and $b_{k}$ with sums *a* and *b*, respectively, where *k* goes from 1 to *K*, then

$$\sum_{i=1}^{K}a_{i}\log\frac{a_{k}}{b_{k}}\leq a\log\frac{a}{b},$$

with equality iff $\frac{a_{k}}{b_{k}}$ are equal for all *i*. By applying this inequality to the above terms in square braces, we have that $\bar{I}\left(Q^{\prime }\right)\leq \bar{I}\left(Q\right)$, with equality iff $\frac{q_{1i}}{p(y_{1})}=\frac{q_{2i}}{p(y_{2})}$ for all *i*. Since $p(y_{1})$ and $p(y_{2})$ are nonzero and independent of *i*, this is true iff column 1 of *Q* is a constant multiple $p\left(y_{1}\right)/p\left(y_{2}\right)$ of column 2. In fact, this also shows that $p(y_{1})$ is a constant multiple of $p(y_{2})$, regardless of the all of the positive input probabilities. □

### 1.2. Back to Our Binary-Input Binary-Output DMC, the *(2,2) Channel*

Restating (1) and following the approach of [13]:

(10) $$\begin{array}{cc} x=P(X=0),\; & \bar{x}=P\left(X=1\right)\;\mathrm{and}\;\mathrm{we}\;\mathrm{define}\;\\ y:=P(Y=0),\; & \;\mathrm{thus}\;\bar{y}=P\left(Y=1\right)\;. \end{array}$$

The above expressions simplify for our DMC under investigation. Using (1) and (2), we have that the distribution of *Y* is

$$\left(y,\bar{y}\right)=\left(x,\bar{x}\right)\left(\begin{array}{cc} a & \bar{a}\\ b & \bar{b} \end{array}\right)=\left(\left(a-b\right)x+b,\;1-\left[(a-b)x+b\right]\right)$$

We now define a differentiable function f(x),x∈[0,1] by

(11) $$f\left(x\right):=\left(a-b\right)x+b=ax+b\bar{x}\;,$$

which gives us

(12) $$\left(y,\bar{y}\right)=\left(f\left(x\right),\bar{f(x)}\right)\;.$$

Thus,

(13) H(Y)=h(y)=h(f(x)).

From (5), we have that

(14) $$\begin{array}{cc} H(Y|X) & =-\left\{x\left[a\log a+\bar{a}\log\bar{a}\right]+\bar{x}\left[b\log b+\bar{b}\log\bar{b}\right]\right\}\\ \; & =x\cdot h\left(a\right)+\bar{x}\cdot h\left(b\right)\;. \end{array}$$

Putting the above together gives us

(15) $$I\left(Y,X\right)=h\left(f\left(x\right)\right)-x\cdot h\left(a\right)-\bar{x}\cdot h\left(b\right)\;.$$

Using (9), we have that the capacity of the *(2,2) channel* is

(16) $$C_{2,2}=\max_{x}I\left(Y,X\right)=\max_{x}\left[h\left(f\left(x\right)\right)-x\cdot h\left(a\right)-\bar{x}\cdot h\left(b\right)\right]\;.$$

So, for the *(2,2) channel*, the capacity calculation boils down to a (not so simple) calculus problem. Silverman [14] was the first to express the closed form result (see also [5,13] and ([Equation (5)] [7]) for derivations and alternate expressions).

(17) $$C_{2,2}\left(a,b\right)=\log\left(2^{\frac{\bar{a}\cdot h\left(b\right)-\bar{b}\cdot h\left(a\right)}{a-b}}+2^{\frac{b\cdot h(a)-a\cdot h(b)}{a-b}}\right)\;,\;\mathrm{where}\;C\left(a,a\right):=0\;,$$

which is a continuous function on the unit square [0,1]×[0,1]. It is trivial to show that capacity is continuous on the unit square without the main diagonal a=b. However, to prove continuity on the entire unit square requires some work and uses the fact that (15) is continuous in *a*, *b*, and *x* see ([Section 2.4] [15]).

**Figure 3:**
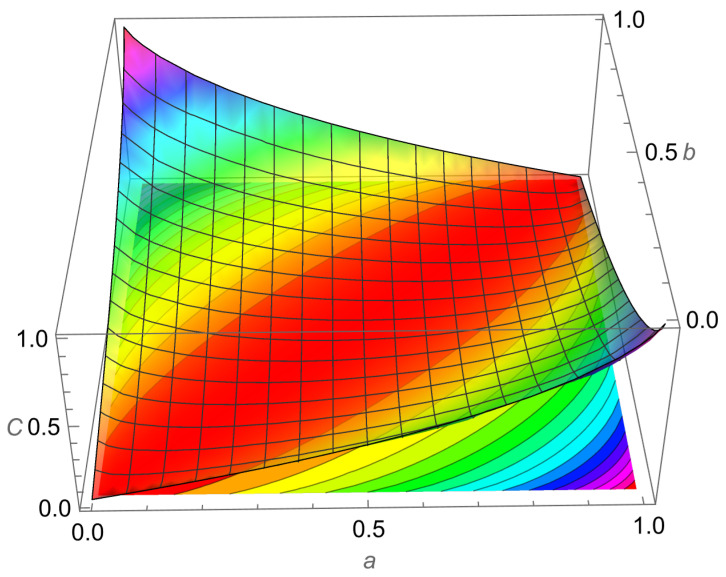
Plot of $C_{2,2}\left(a,b\right)$ along with its level set contours. This figure shows the symmetries (18) about the lines y=x and y=−x+1 as seen by how the countours can be folded onto each other across the two lines. *C* is the capacity.

One can easily show that (see Figure 3)

(18) $$C_{2,2}\left(a,b\right)=C_{2,2}\left(b,a\right)=C_{2,2}\left(\bar{a},\bar{b}\right)\;,$$

by simple algebraic substitution. Additionally, this tells us that $C_{2,2}\left(a,b\right)=C_{2,2}\left(\bar{b},\bar{a}\right)$ also.

$C_{2,2}\left(a,b\right)=C_{2,2}\left(b,a\right)$ is equivalent to capacity being symmetric across the line b=a, and $C_{2,2}\left(a,b\right)=C_{2,2}\left(\bar{b},\bar{a}\right)$ is equivalent to capacity being symmetric when across the line b=−a+1 (simple geometry proves this). This result is illustrated in Figure 3. Thus, capacity has a quadrant of the unit square as its principal domain (see ([Figure 1] [14])).

### 1.3. Power/Fidelity Constraints of $C_{2,2}$

We consider the situation where we attempt to increase the capacity by adjusting the terms *a* and *b*. Ideas like this for a Team’s interdependence, with a different measurement and no mention of information theory, were discussed in [1]. However, the values of a,b are a function of the transmitting environment from $A_{X}$ to $A_{Y}$. If the agents were all-powerful, that could simply adjust *a* to be 1, and *b* to be 0 (or visa versa) to achieve a channel of maximal capacity $C_{2,2}=1$.

#### 1.3.1. Positive Channels

Let us start by considering positive channels [6], that is a>b. Note if a<b, we have a negative channel, and if a=b, we have a 0-capacity channel. Of course, no matter what C(a,b),≥0. However, if we are at a point (a,b), is it better to increase *a*, decrease *b*, or some combination thereof? Implicit in this question is that we stay in the domain of positive channels (under the line b=a).

**Definition** **2**. *We say that we have a power constraint P when we are at the channel given by (a,b) and the most we can adjust the channel is to $\left(a^{\prime },b^{\prime }\right)$ where the standard Euclidean distance (its $l^{2}$ norm) between (a,b) and $\left(a^{\prime },b^{\prime }\right)$ is no more that P.*

In terms of Information Geometry [1], our distance is obtained from the Riemannian metric

(19) $$ds^{2}=da^{2}+db^{2}\;.$$

Of course we can generalize this to a more general metric of the form

(20) $$ds^{2}=E\;da^{2}+F\;da\cdot db+G\;db^{2}\;,$$

which would put us in a non-Euclidean situation. This non-trivial situation may be necessary if *a* and *b* relate differently to various transmission characteristics.

It is shown in ([Theorem 4.9] [6]) that if we restrict ourselves to positive channels, that the capacity increases as *a* increases, and decreases as *b* increases. This result makes physical sense in terms of adding or decreasing noise. Now consider the (closed) disk of radius *r* about the point (a,b), denoted as $D_{r}\left(a,b\right)$. We assume that *r* is small enough so that $D_{r}\left(a,b\right)$ is composed only of positive channels.

**Figure 4:**
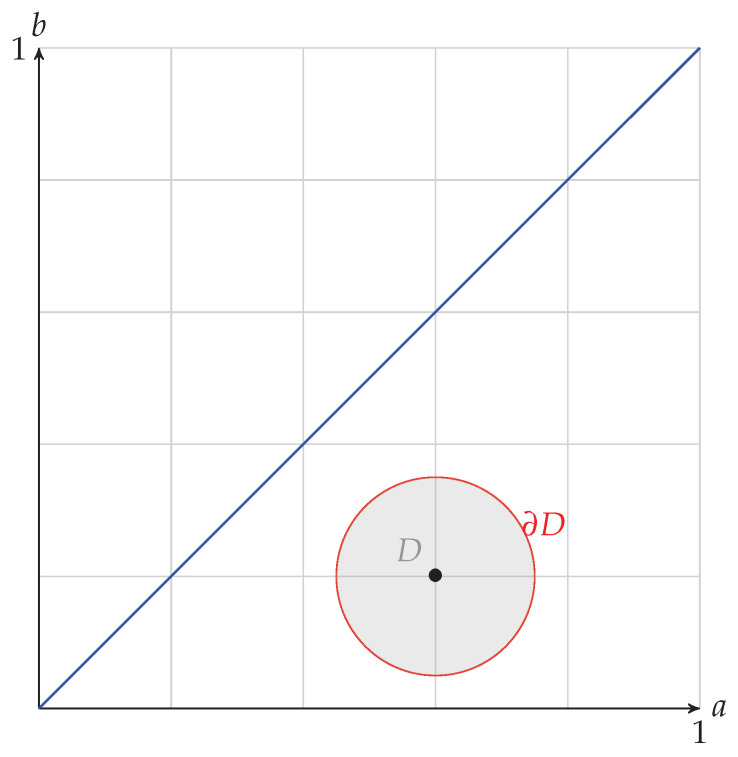
Closed disk *D* of radius 0.15, about the point (0.6,0.2), that consists only of positive channels. The boundary of the disk is the circle ∂D.

**Example** **1**. *We illustrate this situation in Figure 4 by the channels that are in the disk or radius 0.15 about the point (0.6,0.2).*

**Theorem** **1**. *Given a closed disk $D_{r}\left(a,b\right)$ consisting of positive channels, the maximum capacity is achieved and occurs on the boundary circle $\partial D_{r}\left(a,b\right)$.*

**Proof.**  Since $C_{2,2}\left(a,b\right)$ is a continuous function on the compact set $D_{r}\left(a,b\right)$, it is has a maximum denoted as $C_{M}$. Assume that the maximum is achieved at an interior point $\left(a^{\prime },b^{\prime }\right)\in D_{r}\left(a,b\right)$. By ([Theorem 4.9] [6]) we know that increasing $a^{\prime }$ increases capacity, which contradicts $C_{M}$ being achieved at the interior point $\left(a^{\prime },b^{\prime }\right)$. □

We note that the above theorem still holds for non-positive channels by a simple adjustment of the proof.

**Figure 5:**
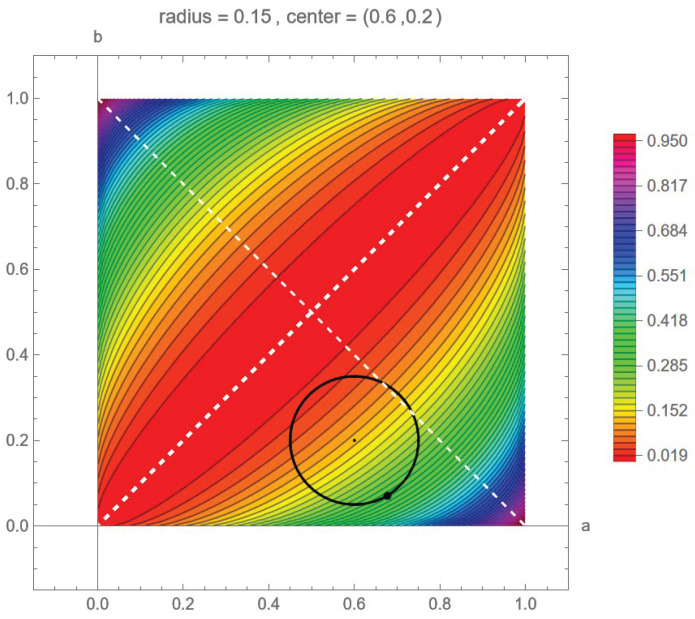
Example 1 illustrated with level sets of capacity with more detail than Figure 4.

**Figure 6:**
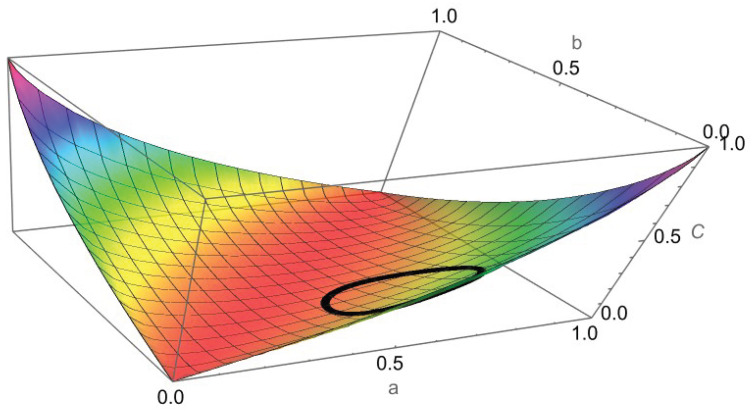
Same as Figure 5, but with a 3D perspective.

Example 1 is illustrated in Figure 4 and is examined again in Figure 5 and Figure 6, where we can see the level sets of $C_{2,2}$ and the surface plot of capacity. Furthermore, numerical calculations show that the maximum of capacity for the closed disk is obtained at the boundary points (0.68,0.07) and has a value of 0.32.

Of course, as the center of the disk and the radius vary, so does the relative position of the point on the circle that capacity is achieved at. What is interesting is that it is not obvious where this point should be. We will explain this further. For a positive channel, increasing *a* brings increased capacity, whereas decreasing *b* results in increased capacity. So, considering our example of the disk centered at (0.6,0.2) with radius 0.15, one might think that this critical point is when *b* is decreased by the amount that *a* is increased—this being the point on the boundary circle at $2\pi -\frac{\pi }{4}=5.50$ radians, which only gives us a capacity of 0.31. However, numerical methods tell us that the actual maximum occurs are 5.25 radians with a value, as noted, of 0.32. Of course, for this example, the difference is not much, but this result is relative to the size of the disk. What is important is that the actual critical point depends on the disk’s position to the two lines b=a and b=1−a. We do note that when the disk is centered on the line b=1−a, that $2\pi -\frac{\pi }{4}$ radians is the correct position for the critical point. One can also see this by examining the capacity level sets in Figure 5.

Of course, we are using an $l^{2}$ metric which has a metric ball of a disk. If, for example, we used an $l^{1}$ metric, the ball would be a square rotated by 45 degrees.

#### 1.3.2. Power

We assume that the transmitting agent $A_{X}$ has adjustable power *P*. This power allows the transmission capabilities of $A_{X}$ to vary. By way of example, say that $A_{X}$ transmits with fidelity a=0.6,b=0.2. Now, $A_{X}$ is given an increase in its transmitting power that allows it to change (a,b) to $\left(a^{\prime },b^{\prime }\right)$ such that the “distance” between the two points is less than *P*. Consider that we use the $L_{2}$ Euclidean norm and set P=0.15. This tells us that all such points $\left(a^{\prime },b^{\prime }\right)$ are in the disk of radius 0.15 about the center (0.6,0.2). We note that this is a rudimentary concept of power. Power helps a transmission when we are restricted to the bottom quarter of this disk and where *a* is increasing (giving more transmission fidelity) and *b* is decreasing (more transmission fidelity—recall that *b* gives us the probability of a 1 going to the opposite symbol 0). However, the conclusion is still the same point that we made and illustrated above.

#### 1.3.3. Results and Discussion

We end this section with a brief summary. We have discussed how one agent can pass Shannon information to another and how changing the transmission characteristics can increase or decrease this information transfer. We have used capacity as our metric for information transfer. Let us now progress to multiple agents. We have also proven some information theoretic properties for the reader (Properties 1 & 2).

In the situation that we discussed in this section where there are two transmitting agents and one receiving agent, we denote the channel as $M_{1}$, which is given by the channel matrix (In this article, we freely identify a channel with its matrix. Furthermore, for a 2×2 channel, we identify the channel as the ordered 2-tuple (a,b) also.) $M_{1}$ described earlier (4). We denote that channel capacity as $C(M_{1})$ which we have analyzed as $C_{2,2}$ in this section.

## 2. Two Transmitting Agents

Say we have two transmitting agents, $A_{X_{1}}$ and $A_{X_{2}}$ acting independently with respect to each other. Assume they have the same transmitting characteristics; that is, the channel matrices are the same. The receiving agent $A_{Y}$ gets symbols from both transmitting agents. How does this impact the information flow to $A_{Y}$?

In our scenario, $A_{X_{1}}$ and $A_{X_{2}}$ both sense the same environment. That is, they both wish to send a 0 or they both wish to send a 1. So, as before, the possible inputs are 0 or 1, but the outputs are of the form

(21) (0,0),(0,1),(1,0),(1,1)

since we are assuming that the noise affects each transmitting agent independently. Keep in mind that both $A_{X_{1}}$ and $A_{X_{2}}$ are both attempting to transmit the same symbol.

The output that $A_{Y}$ uses is given by the random variable *Y*.

$$\begin{array}{cc} \left(0,0\right) & \;\mathrm{is}\;\mathrm{taken}\;\mathrm{to}\;\mathrm{be}\;\mathrm{the}\;\mathrm{symbol}\;Y=O_{0,0}\\ \left(0,1\right) & \;\mathrm{is}\;\mathrm{taken}\;\mathrm{to}\;\mathrm{be}\;\mathrm{the}\;\mathrm{symbol}\;Y=O_{0,1}\\ \left(1,0\right) & \;\mathrm{is}\;\mathrm{taken}\;\mathrm{to}\;\mathrm{be}\;\mathrm{the}\;\mathrm{symbol}\;Y=O_{1,0}\\ \left(1,1\right) & \;\mathrm{is}\;\mathrm{taken}\;\mathrm{to}\;\mathrm{be}\;\mathrm{the}\;\mathrm{symbol}\;Y=O_{1,1}\;. \end{array}$$

We denote $P\left(Y=O_{i,j}\right)=:y_{i,j}$. Our channel matrix is 2×4 and is

$$\begin{array}{ccc} M_{2}\;\;\;\;\; & = & \;\;\;\;\;\left(\begin{array}{cccc} P(Y=O_{0,0}|X=0) & P(Y=O_{0,1}|X=0) & P(Y=O_{1,0}|X=0) & P(Y=O_{1,1}|X=0)\\ P(Y=O_{0,0}|X=1) & P(Y=O_{0,1}|X=1) & P(Y=O_{1,0}|X=1) & P(Y=O_{1,1}|X=1) \end{array}\right)\\ & = & \;\;\;\;\left(\begin{array}{cccc} a^{2} & a\bar{a} & \bar{a}a & {\bar{a}}^{2}\\ b^{2} & b\bar{b} & \bar{b}b & {\bar{b}}^{2} \end{array}\right)\;. \end{array}$$

We note that the second and third columns of the above channel matrix are identical. This has implications for the mutual information and, of course, the capacity of the channel.

Let us look at this in more generality. Say we have two channel matrices

$$M^{3}=\left(\begin{array}{ccc} \alpha & 2\epsilon & \delta \\ \beta & 2\gamma & \phi \end{array}\right)\;\;\;\mathrm{and}\;\;\;\;M^{4}=\left(\begin{array}{cccc} \alpha & \epsilon & \epsilon & \delta \\ \beta & \gamma & \gamma & \phi \end{array}\right)\;.$$

Both channels have the same input random variable *X* as above. The output random variables are $Y^{3}$ and $Y^{4}$, respectively.

Let us consider the $M^{3}$ channel first. $Y^{3}$ has probability values $y_{i}:=P\left(Y^{3}=i\right)$ as follows

$$\left(y_{1},y_{2},y_{3}\right)=\left(\alpha x+\beta \bar{x},2\epsilon x+2\gamma \bar{x},\delta x+\phi \bar{x}\right)\;.\;\mathrm{So},\;\;\;\;H\left(Y^{3}\right)$$

$$=-\left[\left(\alpha x+\beta \bar{x}\right)\log\left(\alpha x+\beta \bar{x}\right)+\left(2\epsilon x+2\gamma \bar{x}\right)\log\left(2\epsilon x+2\gamma \bar{x}\right)+\left(\delta x+\phi \bar{x}\right)\log\left(\delta x+\phi \bar{x}\right)\right]\;,$$

(22) $$\begin{array}{cc} H(Y^{3}|X) & =-x\left[\alpha \log(\alpha )+2\epsilon \log(2\epsilon )+\delta \log(\delta )\right]\\ & -\bar{x}\left[\beta \log(\beta )+2\gamma \log(2\gamma )+\phi \log(\phi )\right]\;. \end{array}$$

The mutual information is I(Y,X)=H(Y)−H(Y|X). We expand the mutual information into the sum of two functions. The first function is from the first and last columns, and the second function is from the middle column. That is

$$I\left(Y^{3},X\right)=F_{1}^{3}\left(\alpha ,\beta ,\delta ,\phi ,x\right)+F_{2}^{3}\left(\epsilon ,\gamma ,x\right)\;,\;\mathrm{where}\;\;$$

$$\begin{array}{ccc} F_{2}^{3} & = & -2\epsilon x\log\left(2\epsilon x+2\gamma \bar{x}\right)-2\gamma \bar{x}\log\left(2\epsilon x+2\gamma \bar{x}\right)+2\epsilon x\log\left(2\epsilon \right)+2\gamma \bar{x}\log\left(2\gamma \right)\\ & = & 2\epsilon x\log\left(\frac{2\epsilon }{2\epsilon x+2\gamma \bar{x}}\right)+2\gamma \bar{x}\log\left(\frac{2\gamma }{2\epsilon x+2\gamma \bar{x}}\right)\\ & = & 2\epsilon x\log\left(\frac{\epsilon }{\epsilon x+\gamma \bar{x}}\right)+2\gamma \bar{x}\log\left(\frac{\gamma }{\epsilon x+\gamma \bar{x}}\right)\;. \end{array}$$

Now let us consider the $M^{4}$ channel. As above

$$\left(y_{1},y_{2},y_{3},y_{4}\right)=\left(\alpha x+\beta \bar{x},\epsilon x+\gamma \bar{x},\epsilon x+\gamma \bar{x},\delta x+\phi \bar{x}\right)\;.$$

$$\begin{array}{cc} H(Y^{4}) & =-[\left(\alpha x+\beta \bar{x}\right)\log\left(\alpha x+\beta \bar{x}\right)+\left(\epsilon x+\gamma \bar{x}\right)\log\left(\epsilon x+\gamma \bar{x}\right) \end{array}$$

(23) $$\begin{array}{cc} \; & +\left(\epsilon x+\gamma \bar{x}\right)\log\left(\epsilon x+\gamma \bar{x}\right)+\left(\delta x+\phi \bar{x}\right)\log\left(\delta x+\phi \bar{x}\right)]\\ \; & =-[\left(\alpha x+\beta \bar{x}\right)\log\left(\alpha x+\beta \bar{x}\right)+2\left(\epsilon x+\gamma \bar{x}\right)\log\left(\epsilon x+\gamma \bar{x}\right) \end{array}$$

(24) $$\begin{array}{cc} \; & +\left(\delta x+\phi \bar{x}\right)\log\left(\delta x+\phi \bar{x}\right)]\;. \end{array}$$

$$\begin{array}{cc} H(Y^{4}|X) & =-x\left[\alpha \log(\alpha )+\epsilon \log(\epsilon )+\epsilon \log(\epsilon )+\delta \log(\delta )\right] \end{array}$$

(25) $$\begin{array}{cc} & -\bar{x}\left[\beta \log(\beta )+\gamma \log(\gamma )+\gamma \log(\gamma )+\phi \log(\phi )\right]\\ \; & =-x\left[\alpha \log(\alpha )+2\epsilon \log(\epsilon )+\delta \log(\delta )\right] \end{array}$$

(26) $$\begin{array}{cc} & -\bar{x}\left[\beta \log(\beta )+2\gamma \log(\gamma )+\phi \log(\phi )\right]\;. \end{array}$$

As above we expressthe mutual information as

$$I\left(Y^{3},X\right)=F_{1}^{3}\left(\alpha ,\beta ,\delta ,\phi ,x\right)+F_{2}^{3}\left(\epsilon ,\gamma ,x\right)$$

and we have that

$$\begin{array}{ccc} F_{2}^{4} & = & -2\epsilon x\log\left(\epsilon x+\gamma \bar{x}\right)-2\gamma \bar{x}\log\left(\epsilon x+\gamma \bar{x}\right)+2\epsilon x\log\left(\epsilon \right)+2\gamma \bar{x}\log\left(\gamma \right)\\ & = & 2\epsilon x\log\left(\frac{\epsilon }{\epsilon x+\gamma \bar{x}}\right)+2\gamma \bar{x}\log\left(\frac{\gamma }{\epsilon x+\gamma \bar{x}}\right)=F_{2}^{3}\;. \end{array}$$

A quick inspection tells us that $F_{1}^{4}=F_{1}^{3}$; thus, the mutual information of both channels is the same. This result is not surprising because if we combine output symbols where the channel matrix has identical rows, we lose nothing as far as the output information is concerned—there is no extra value in looking at the output symbols separately. This makes sense, and is also what our mathematics have shown.

Let us keep in mind that we wish to find $C(M_{2})$, the capacity of the Shannon channel when there are two transmitting agents. (To keep our notation consistent, C(a,b) is the capacity given by the corresponding 2×2 channel matrix as in (4), whereas C(∗) is the capacity of the channel given by *).

**Theorem** **2**. *$C\left(M_{2}\right)\geq C\left(M_{1}\right)$.*

**Proof.**  $M_{2}$ has four output symbols which are in essence 2-vectors. We ignore the second component of the vector. Therefore, we collapse the first and third symbol to *a*, and the second and fourth to $\bar{a}$. This results in $M_{1}$, and since using more output symbols never lowers capacity, by Property 2 (also, a code that works for $M_{1}$ works for $M_{2}$ as well by collapsing the symbols), we are done. (Later in the paper we do better than this result with Corollary 1 to Theorem 6.) □

We now form another channel related to what we discussed above. Say now that the receiving agent receives the symbols without any order. Therefore, instead of a 2-vector, the output is one of the three multisets [0,0],[1,0],[1,1] with

$$P\left(Y=\left[0,0\right]\right)=a^{2},P\left(Y=\left[1,0\right]\right)=2\bar{a}a,P\left(Y=\left[1,1\right]\right)={\bar{a}}^{2}\;.$$

We call this channel $M_{2-}$, and its channel matrix is

$$M_{2-}=\left(\begin{array}{ccc} a^{2} & 2\bar{a}a & {\bar{a}}^{2}\\ b^{2} & 2\bar{b}b & {\bar{b}}^{2} \end{array}\right)\;.$$

From what we discussed above with $M^{4}$ and $M^{3}$, we see that

**Theorem** **3**.
$$C\left(M_{2-}\right)=C\left(M_{2}\right)\;.$$

**Figure 7:**
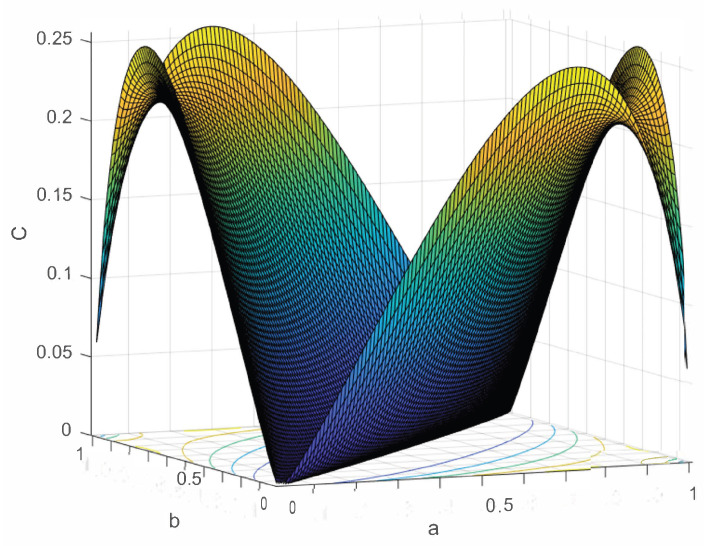
The plot $C\left(M_{2}\right)-C\left(M_{1}\right)$, of course the *C* axis is now measuring the difference in the capacities (in units of bits per *t*).

Let us examine the bounds in Theorem 1 above. We will see that, not surprisingly except for special cases, $C\left(M_{2}\right)>C\left(M_{1}\right)$. Figure 7 is a plot of $C\left(M_{2}\right)-C\left(M_{1}\right)$ as a function of (a,b).

From Figure 7, we see that except for the line b=a (where both channels $M_{1}$ and $M_{2}$ have 0 capacity), and at (a,b)=(1,0) or (a,b)=(0,1) (where both channels have capacity 1), that $C\left(M_{2}\right)>C\left(M_{1}\right)$. We note that for $M_{2}$ and the other higher dimensional channels that we will discuss, there is to our knowledge no closed form as there is for $M_{1}$. Therefore, for our calculations of capacity, we rely upon numerical results from the Blahut-Arimoto capacity algorithm [16,17].

### Results and Discussion

In this section, we have laid the groundwork for *n* transmitting agents. We derived some capacity results. We concentrated on the effects of going from 1 to 2 transmitting agents. What happens as we go to three or more transmitting agents?

## 3. Multiple Transmitting Agents

We have the canonical representation for the channel of *n* transmitting agents, and we denote this canonical channel matrix as $M_{\underline{n}}$, which is formed by taking the output of channel $M_{\underline{n-1}}$ (Note, due to the simplicity of the construction for “small” channels, we have that $M_{\underline{1}}=M_{1},M_{\underline{2}}=M_{2}$.) and adding a 0 or a 1 to it. For $M_{\underline{3}}$ this results in

$$M_{\underline{3}}=\left(\begin{array}{cccccccc} a^{3} & a^{2}\bar{a} & a^{2}\bar{a} & a{\bar{a}}^{2} & a^{2}\bar{a} & a{\bar{a}}^{2} & a{\bar{a}}^{2} & {\bar{a}}^{3}\\ b^{3} & b^{2}\bar{b} & b^{2}\bar{b} & b{\bar{b}}^{2} & b^{2}\bar{b} & b{\bar{b}}^{2} & b{\bar{b}}^{2} & {\bar{b}}^{3} \end{array}\right)\;.$$

This comes from taking the output for two agents as given in canonical form by (21) and extending it to

(0,0,0),(0,0,1),(0,1,0),(0,1,1),(1,0,0),(1,0,1),(1,1,0),(1,1,1).

**Theorem** **4**. *Rearranging outputs/columns of a channel matrix does not affect capacity.*

**Proof.**  By looking at the expression for mutual information, we see that changing the order of arithmetic operations leaves it unchanged. This result follows, since capacity is the maximum of mutual information. □

Therefore, we can permute the columns of $M_{\underline{n}}$ and obtain a new matrix $M_{n}$, which has the same capacity, that is $C\left(M_{n}\right)=C\left(M_{\underline{n}}\right)$, and is given below.

(27) $$M_{n}=\left(\begin{array}{ccccccccc} a^{n} & a^{n-1}\bar{a} & \ldots & a^{n-1}\bar{a} & a^{n-2}{\bar{a}}^{2} & \ldots & a^{n-1}\bar{a} & \ldots & {\bar{a}}^{n}\\ b^{n} & b^{n-1}\bar{b} & \ldots & b^{n-1}\bar{b} & b^{n-2}{\bar{b}}^{2} & \ldots & b^{n-1}\bar{b} & \ldots & {\bar{b}}^{n} \end{array}\right)\;.$$

Look at the above theorem in terms of the columns of $M_{n}$. Let us use $M_{3}$ as an example.

(28) $$M_{3}=\left(\begin{array}{cccccccc} a^{3} & a^{2}\bar{a} & a^{2}\bar{a} & a^{2}\bar{a} & a{\bar{a}}^{2} & a{\bar{a}}^{2} & a{\bar{a}}^{2} & a^{3}\\ b^{3} & b^{2}\bar{b} & b^{2}\bar{a} & b^{2}\bar{b} & b{\bar{b}}^{2} & b{\bar{b}}^{2} & b{\bar{b}}^{2} & b^{3} \end{array}\right)\;.$$

Collapsing the output in this situation is equivalent to interchanging the 4th and 5th columns (which does not change capacity) and forming the matrix $M_{3c}$.

(29) $$M_{3c}=\left(\begin{array}{cccccccc} a^{3} & a^{2}\bar{a} & a^{2}\bar{a} & a{\bar{a}}^{2} & a^{2}\bar{a} & a{\bar{a}}^{2} & a{\bar{a}}^{2} & a^{3}\\ b^{3} & b^{2}\bar{b} & b^{2}\bar{a} & b{\bar{b}}^{2} & b^{2}\bar{b} & b{\bar{b}}^{2} & b{\bar{b}}^{2} & b^{3} \end{array}\right)\;.$$

As above when we looked at $M^{3}$ and $M_{4}$, we see that we may form the channel where we identify output symbols with the same conditional probabilities for both inputs. This give us the channel $M_{n}-$, where

(30) Mn−=annan−1a¯n2an−2a¯2…naa¯n−1a¯nbnnbn−1b¯n2bn−2b¯2…nbb¯n−1b¯n.

**Theorem** **5**.
$C\left(M_{n}\right)=C\left(M_{n-}\right)$

**Proof.**  As above for $M_{2}$ in Theorem 3, or we can just use Property 2 repeatedly. □

**Figure 8:**
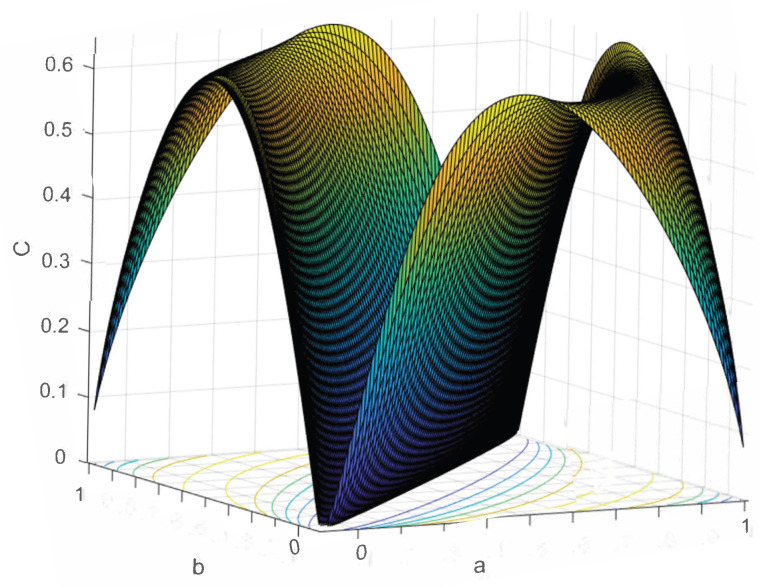
$C\left(M_{8}\right)-C\left(M_{1}\right)$.

The reason we introduce $M_{n-}$ is that it is a cleaner way to express the channel, and the calculations are simpler than that of $M_{n}$. For example, $M_{8}$ is a 2×256 matrix, whereas $M_{8-}$ is a 2×9 matrix. This obviously makes the coding issues easier. Now we examine Figure 8, which is the difference between $C(M_{8})$ and $C(M_{1})$.

When we compare Figure 8 to Figure 7, we easily see that $C(M_{n})$ grows, except for the endpoints and the line b=a (which stay at 0) as n grows.

*Nota Bene* We now look at the prior illustrative results in terms of a more general encompassing theory. We included much of Section 2 so that the reader who is not familiar with some of the “tricks” will have a feel for why the more general results hold.

**Theorem** **6**. *$C\left(M_{n+1}\right)\geq C\left(M_{n}\right)$ for any positive integer n.*

**Proof**. (The proof is the same as for the above when n=1.) $M_{n}$ can be obtained from $M_{n+1}$ by combining certain columns together; the result follows from Property 2. □

**Corollary** **1**. *$C\left(M_{n+1}\right)>C\left(M_{n}\right)$, except for (1,0) and (0,1) where they both have capacity 1, and the line b=a where they both have capacity 0.*

**Proof.**  We show the proof in three steps.

1. If a=b, $C\left(M_{n}\right)=C\left(M_{n+1}\right)=0$ since the rows are identical. In this case, it is trivial to show that H(Y)=H(Y|X) (the output has no idea what the channel input was). One can see this by the fact that $x\cdot a^{q}{\bar{a}}^{n-q}+\bar{x}\cdot a^{q}{\bar{a}}^{n-q}=a^{q}{\bar{a}}^{n-q}$. In short, the capacities are equal.
2. If (a,b)=(1,0) or (a,b)=(0,1), both $M_{n}$ and $M_{n+1}$ are both the 2×2 identity matrix with zero columns added in; hence, $C\left(M_{n}\right)=C\left(M_{n-1}\right)=1$. In short, the channel capacities are equal.
3. Now, excluding the special cases where a=b, (a,b)=(1,0), or (a,b)=(0,1), by Property 2, we only have to show that here are two combined columns that are not multiples of each other.

By excluding the special cases, we cannot use the endpoints of the unit square; therefore, *a* or *b* must be in (0,1). WLOG, we *assume* that 0<a<1.

Consider a generic column of $M_{n}$; it is of the form $c=\left(\begin{array}{c} a^{e}{\bar{a}}^{n-e}\\ b^{e}{\bar{b}}^{n-e} \end{array}\right),e\in \left\{0,\ldots ,n\right\}$. By construction, $M_{n+1}$ has two columns, $c_{1}=\left(\begin{array}{c} a\cdot a^{e}{\bar{a}}^{n-e}\\ b\cdot b^{e}{\bar{b}}^{n-e} \end{array}\right)$ and $c_{2}=\left(\begin{array}{c} \bar{a}\cdot a^{e}{\bar{a}}^{n-e}\\ \bar{b}\cdot b^{e}{\bar{b}}^{n-e} \end{array}\right),$ that when combined result in column *c*. If $c_{1}$ is not a constant multiple of $c_{2}$, we will have shown that $C\left(M_{n+a}\right)>C\left(M_{n}\right)$. Assume the opposite—that is, $c_{1}=k\cdot c_{2}$; since neither *a* or $\bar{a}$ is 0 we have that $a=k\bar{a}$. Then $a=k\bar{a}$ is equivalent to $a=\frac{k}{k+1},k\neq 0$. We now have three cases for b.

- b=0. In this case, $\bar{b}=1$ and we only look at the last column of $M_{n}$, so we let $c=\left(\begin{array}{c} {\bar{a}}^{n}\\ {\bar{b}}^{n} \end{array}\right)=\left(\begin{array}{c} {\bar{a}}^{n}\\ 1\; \end{array}\right).$ Since we are assuming that $c_{1}=k\cdot c_{2}$, we have that
  $0=0\cdot 1=b\cdot 1=k\cdot \bar{b}\cdot 1=k$, which is impossible.
- b=1. Using the same argument as above, just replace the last column of $M_{n}$ with the first. So again, it is impossible that the columns are multiples.
- 0<b<1. As above for *a*, we also have that $b=\frac{k}{k+1}$. This tells us that a=b which has been ruled out.

Thus, we have shown the existence of two columns of $M_{n+1}$ that are not multiples of each other and combine them into a column of $M_{n}$. □

**Theorem** **7**. *$\lim_{n\to \infty }C\left(M_{n}\right)=1$, except for when b=a, and in that case, the channel capacity is 0.*

**Proof.**  WLOG, we assume a>b. We can do this because of the constraint a≠b and the fact that the rows of a channel matrix can be interchanged without affecting its capacity. Take a positive $\varepsilon <<\frac{a-b}{2}$ be fixed. For a large enough *N*, we can always find a rational number m(n) for any n>N such that $\bar{a}+\varepsilon <m<\bar{b}-\varepsilon <1$ and nm∈Z. (The ε padding prevents *m* from converging to $\bar{a}$ or $\bar{b}$). This result is guaranteed to exist for sufficiently large *N*.

Given 0≤b<a≤1, let $x=\bar{a}+\varepsilon ,y=\bar{b}-\varepsilon$, giving us 0≤x<y≤1. Certainly there exists a positive integer *N* such that 1/N<y−x. Therefore, for any integer n≥N, we have that 1/n<y−x. Consider (x,y) as a sub-interval of [0,1]. For any n≥N, consider the largest integer *W* such that W(1/n)≤x. Look at (W+1)(1/n); by the definition of *W*, this must be greater than *x*. However, since 1/n<y−x, we have that (W+1)(1/n)<y. We let m=(W+1)(1/n). Keep in mind two characteristics of *m* as a function of *n*:

1. Since *W* is an integer, mn∈Z, and,
2. mn<n, since m<1.

Let $M_{n}^{\prime }$ be the channel matrix $M_{n-}$, but modified as follows: all outputs $y_{k}$ for k≤mn are combined into $y_{0}^{\prime }$, and all of the other outputs are combined into $y_{1}^{\prime }$. The channel matrix then looks like this:

$$M_{n}^{\prime }=\left(\begin{array}{cc} P(y_{0}^{\prime }|x_{0}) & P(y_{1}^{\prime }|x_{0})\\ P(y_{0}^{\prime }|x_{1}) & P(y_{1}^{\prime }|x_{1}) \end{array}\right),$$

where

$$\left(Y=y_{0}^{\prime }\right)=\left(Y=y_{0}\right)\cup \left(Y=y_{1}\right)\cup \ldots \cup \left(Y=y_{mn}\right)⊊\left(Y=y_{o}\right)\cup \ldots \cup \left(Y=y_{n}\right)\;\mathrm{and}\;$$

P(y0′|x0)=∑i=0mnP(yi|x0),withP(yi|x0)=nian−ia¯i.

(Keep in mind that we are dealing with the binomial random variable $S_{n}$, where *i* is the number of successes in *n* Bernoulli trials, with the probability of success $\bar{a}$, P(Sn=i)=nian−ia¯i).

∴P(y0′|x0)=∑i=0mnnian−ia¯i.

If we let Φ(x) be the cumulative standard normal distribution function, the De-Moivre Laplace limit theorem [18] states that (when we take c,d as integers)

$$\begin{array}{c} P\left(c<\frac{S_{n}-n\bar{a}}{\sqrt{na\bar{a}}}<d\right)\to \Phi \left(d\right)-\Phi \left(c\right)\;\mathrm{as}\;n\to \infty ;\;\mathrm{thus},\;\\ P\left(\frac{c-\bar{a}}{\sqrt{na\bar{a}}}<\frac{S_{n}-n\bar{a}}{\sqrt{na\bar{a}}}<\frac{d-\bar{a}}{\sqrt{na\bar{a}}}\right)\to \Phi \left(\frac{d-\bar{a}}{\sqrt{na\bar{a}}}\right)-\Phi \left(\frac{c-\bar{a}}{\sqrt{na\bar{a}}}\right)\;\mathrm{as}\;n\to \infty ,\;\mathrm{and}\;\\ P\left(c\leq S_{n}\leq d\right)\to \Phi \left(\frac{d-\bar{a}}{\sqrt{na\bar{a}}}\right)-\Phi \left(\frac{c-\bar{a}}{\sqrt{na\bar{a}}}\right)\;\mathrm{as}\;n\to \infty \;. \end{array}$$

This step leaves us with

(31) ∑i=cdnian−ia¯i→Φd−na¯naa¯−Φc−na¯naa¯asn→∞.

Thus, the De-Moivre Laplace limit theorem gives us (with c=0,d=mn):

$$\begin{array}{cc} \lim_{n\to \infty }P\left(y_{0}^{\prime }|x_{0}\right)\; & =\lim_{n\to \infty }\left[\Phi \left(\frac{mn-n\bar{a}}{\sqrt{na\bar{a}}}\right)-\Phi \left(\frac{-n\bar{a}}{\sqrt{na\bar{a}}}\right)\right]\\ \; & =\lim_{n\to \infty }\Phi \left(\sqrt{n}\frac{m-\bar{a}}{\sqrt{a\bar{a}}}\right)-\lim_{n\to \infty }\Phi \left(\sqrt{n}\frac{-\bar{a}}{\sqrt{a\bar{a}}}\right)\;. \end{array}$$

Since *a* and $\bar{a}$ are positive, then $-\frac{\bar{a}}{\sqrt{a\bar{a}}}$ is negative, giving

$$\lim_{n\to \infty }\sqrt{n}\frac{-\bar{a}}{\sqrt{a\bar{a}}}=-\infty ,\;\mathrm{and}\;$$

$$\lim_{n\to \infty }\Phi \left(\sqrt{n}\frac{-\bar{a}}{\sqrt{a\bar{a}}}\right)=0\;.$$

If $m<\bar{a}$, then $\frac{m-\bar{a}}{\sqrt{a\bar{a}}}$ is negative. However, if $m>\bar{a}$, it is positive, giving (Even though *m* changes as *n* changes, the value of $\sqrt{n}\frac{m-\bar{a}}{\sqrt{a\bar{a}}}$ remains greater than or equal to $\sqrt{n}\frac{\varepsilon }{\sqrt{a\bar{a}}}$ for $m>\bar{a}+\varepsilon$. Since $\sqrt{n}\frac{\varepsilon }{\sqrt{a\bar{a}}}$ approaches *∞*, so does $\sqrt{n}\frac{m-\bar{a}}{\sqrt{a\bar{a}}}$. The same logic can also be used for the $m<\bar{a}-\varepsilon$ case.)

$$\lim_{n\to \infty }\sqrt{n}\frac{m-\bar{a}}{\sqrt{a\bar{a}}}=\left\{\begin{array}{cc} -\infty & \;\mathrm{if}\;m<\bar{a}-\varepsilon \\ \infty & \;\mathrm{if}\;m>\bar{a}+\varepsilon \;;\;\mathrm{and}\; \end{array}\right.$$

$$\lim_{n\to \infty }\Phi \left(\sqrt{n}\frac{m-\bar{a}}{\sqrt{a\bar{a}}}\right)=\left\{\begin{array}{cc} 0 & \;\mathrm{if}\;m<\bar{a}-\varepsilon \\ 1 & \;\mathrm{if}\;m>\bar{a}+\varepsilon \end{array}\right.$$

$$∴\;\lim_{n\to \infty }P\left(y_{0}^{\prime }|x_{0}\right)=1-0=1\;.$$

Thus, we have that

$$\lim_{n\to \infty }P\left(y_{1}^{\prime }|x_{0}\right)=0\;.$$

$P(y_{0}^{\prime }|x_{1})$ beahves the same, but with *a* replaced by *b*. Since $\bar{a}+\varepsilon <m<\bar{b}-\varepsilon$, then the $\lim_{n\to \infty }P\left(y_{0}^{\prime }|x_{0}\right)=1$ and $\lim_{n\to \infty }P\left(y_{0}^{\prime }|x_{1}\right)=0$; thus,

$$\lim_{n\to \infty }M_{n}^{\prime }=\left(\begin{array}{cc} 1 & 0\\ 0 & 1 \end{array}\right).$$

which has a channel capacity of 1. Since $M_{n}^{\prime }$ was formed by combining the outputs of $M_{n}$, then $C\left(M_{n}^{\prime }\right)\leq C\left(M_{n}\right)\leq 1$. Therefore, by the squeeze theorem, $\lim_{n\to \infty }C\left(M_{n}\right)=1$. □

### Results and Discussion

The theorems presented in this section shows what happens as the number of transmitters grows. The ultimate result of this section was Theorem 7, which used a rather non-trivial application of the Central Limit Theorem. At this point, the seemingly obvious but difficult result that we proved, i.e., that as the number of transmitting agents grows, so does the reliability of the channel in terms of its capacity. This result, of course, is in line with the similar result that if we have a code that consisted of repeating a symbol many times the error rate is small (the transmission rate may be low, but this does not apply to our agent examples).

## 4. Non-Identical Transmitting Agents

In a shift, say we start with only two transmitting agents, but their noise characteristics are different. Of course, keep in mind that in this situation, we have assumed that there is a master transmitter using the *X* agent to communicate with *Y*. The master transmitter picks the input symbols and the transmitting agents do their best to communicate by forming one encompassing Shannon channel. We have shown above that, if all of the agents share the same assumption for (a,b), the channel capacity increases as the number of agents increase. However, what happens if the (a,b) are different for the various agents? Are we better off only using a subset of agents, or is it still best to use as many agents as possible? We partially answer those questions below.

Let $M_{1}^{1}$ be the channel matrix for agent 1, and $M_{1}^{2}$ be the channel matrix for agent 2.

$$M_{1}^{1}=\left(\begin{array}{cc} a & \bar{a}\\ b & \bar{b} \end{array}\right)\;,$$

$$M_{1}^{2}=\left(\begin{array}{cc} c & \bar{c}\\ d & \bar{d} \end{array}\right)\;.$$

The output is such that the receiving agent uses the ordering of agent 1 first, then agent 2. If the agents wish to send a signal of 0, the possible outputs, expressed via their probabilities, are

$$\begin{array}{cc} P(0,0) & =ac\\ P(0,1) & =a\bar{c}\\ P(1,0) & =\bar{a}c\\ P(1,1) & =\bar{a}\bar{c} \end{array}$$

If the agents wish to send a signal of 1 instead, we have

$$\begin{array}{cc} P(0,0) & =bd\\ P(0,1) & =b\bar{d}\\ P(1,0) & =\bar{b}d\\ P(1,1) & =\bar{b}\bar{d}\;. \end{array}$$

This gives us a combined channel matrix for both agents who are transmitting as $M_{2}^{1,2}$, where

(32) $$M_{2}^{1,2}=\left(\begin{array}{cccc} ac & a\bar{c} & \bar{a}c & \bar{a}\bar{c}\\ bd & b\bar{d} & \bar{b}d & \bar{b}\bar{d} \end{array}\right)\;.$$

We use our own notation to express the above channel as the tensor product,

(a,b)⊗(c,d).

We know, by Property 2, that collapsing output symbols does not increase capacity. However, if we collapse $y_{1}$ and $y_{2}$ into $y_{1^{\prime }}$ and $y_{3}$ and $y_{4}$ into $y_{2^{\prime }}$, we have a channel matrix of $M_{2^{\prime }}^{1,2}$:

$$M_{2^{\prime }}^{1,2}=\left(\begin{array}{cc} ac+a\bar{c} & \bar{a}c+\bar{a}\bar{c}\\ bd+b\bar{d} & \bar{b}d+\bar{b}\bar{d} \end{array}\right)=\left(\begin{array}{cc} a & \bar{a}\\ b & \bar{b} \end{array}\right)\;.$$

Thus, $C\left(M_{2}^{1,2}\right)\geq C\left(M_{2^{\prime }}^{1,2}\right)=C\left(M_{1}^{1}\right)$.

Now let us combine the first and third outputs of $M_{2}^{1,2}$ into $y_{1^{\prime \prime }}$ and the second and fourth outputs into $y_{2^{\prime \prime }}$. This gives us a channel matrix $M_{2^{\prime \prime }}^{1,2}$.

$$M_{2^{\prime \prime }}^{1,2}=\left(\begin{array}{cc} ac+\bar{a}c & a\bar{c}+\bar{a}\bar{c}\\ bd+\bar{b}d & b\bar{d}+\bar{b}\bar{d} \end{array}\right)=\left(\begin{array}{cc} c & \bar{c}\\ d & \bar{d} \end{array}\right)\;.$$

Thus, $C\left(M_{2}^{1,2}\right)\geq C\left(M_{2^{\prime \prime }}^{1,2}\right)=C\left(M_{1}^{2}\right)$. This result leads us to the next theorem:

**Theorem** **8**. *As the number of agents increase, no matter if they have different channel noises, the total channel capacity is non-decreasing.*

**Proof.**  In the above discussion we have show that

$$\begin{array}{c} C\left(M_{2}^{1,2}\right)\geq C\left(M_{2^{\prime }}^{1,2}\right)=C\left(M_{1}^{1}\right)\\ C\left(M_{2}^{1,2}\right)\geq C\left(M_{2^{\prime \prime }}^{1,2}\right)=C\left(M_{1}^{2}\right). \end{array}$$

Therefore, by repeating the same argument we see that as we add extra agents the capacity can never decrease. □

In fact, as before when the agents had identical characteristics, the channel capacity, except for special cases (dependent columns, a capacity 0 or 1, etc.), is greater than that for separate agents. One can see this by examining the channel matrix—if you unpack the outputs and find that the statistics are different, extra information is learned. Let us now look at the special case of combining a channel with a 0-channel.

**Theorem** **9**. *For any zero channel given by (e,e),e∈[0,1], we find that*

$$C\left((a,b)⊗(e,e)\right)=C\left(a,b\right)\;.$$

**Proof.**  If we can show that the mutual information of (a,b)⊗(e,e) is given by (15), we are done. The channel matrix for this situation is

$$\left(\begin{array}{cccc} ae & a\bar{e} & \bar{a}e & \bar{a}\bar{e}\\ be & b\bar{e} & \bar{b}e & \bar{b}\bar{e} \end{array}\right)\;.$$

Let $u:=ax+b\bar{x}$, and we find that $\bar{u}=\bar{a}x+\bar{b}\bar{x}$. Further,

$$\begin{array}{ccc} Y=(y_{1},y_{2},y_{3},y_{4}) & = & \left(aex+be\bar{x},a\bar{e}x+b\bar{e}\bar{x},\bar{a}ex+\bar{b}e\bar{x},\bar{a}\bar{e}x+\bar{b}\bar{e}\bar{x}\right)\\ & = & (ue,u\bar{e},\bar{u}e,\bar{u}\bar{e})\;, \end{array}$$

$$\begin{array}{ccc} H(Y) & = & -\left[ue\log\left(ue\right)+u\bar{e}\log\left(u\bar{e}\right)+\bar{u}e\log\left(\bar{u}e\right)+\bar{u}\bar{e}\log\left(\bar{u}\bar{e}\right)\right]\\ & = & -\left[ue\left(\log\left(u\right)+\log\left(e\right)\right)+u\bar{e}\left(\log\left(u\right)+\log\left(\bar{e}\right)\right)\right.\\ & & \left.+\bar{u}e\left(\log\left(\bar{u}\right)+\log\left(e\right)\right)+\bar{u}\bar{e}\left(\log\left(\bar{u}\right)+\log\left(\bar{e}\right)\right)\right]\\ & = & -\left[u\log\left(u\right)+\bar{u}\log\bar{u}+e\log\left(e\right)+\bar{e}\log\left(\bar{e}\right)\right]\\ & = & h(u)+h(e)\;,\;\mathrm{and}\; \end{array}$$

$$\begin{array}{ccc} H(Y|X) & = & -x\left[ae\log\left(ae\right)+a\bar{e}\log\left(a\bar{e}\right)+\bar{a}e\log\left(\bar{a}e\right)+\bar{a}\bar{e}\log\left(\bar{a}\bar{e}\right)\right]\\ & & -\bar{x}\left[be\log\left(be\right)+b\bar{e}\log\left(b\bar{e}\right)+\bar{b}e\log\left(\bar{b}e\right)+\bar{b}\bar{e}\log\left(\bar{b}\bar{e}\right)\right]\;. \end{array}$$

Now again using the log of a product as the sum of the logs, then grouping like log terms, this results in

$$H\left(Y|X\right)=x\left[h(a)+h(e)\right]+\bar{x}\left[h(b)+h(e)\right]=x\cdot h\left(a\right)+\bar{x}\cdot \left(b\right)+h\left(e\right)\;,$$

and we see that $\;H\left(Y\right)-H\left(Y|X\right)=h\left(ax+b\bar{x}\right)-x\cdot h\left(a\right)-\bar{x}\cdot h\left(b\right)\;.$ □

### Results and Discussion

In this section, we showed what happens when two transmitting agents with different noise characteristics are used. Our important result was that as the number of agents increase, no matter if they have different channel noises, the total channel capacity is non-decreasing. As with many of our results it relied upon the algebra of mutual information giving common sense answers. However, without proofs we just have intuition to rely upon.

## 5. Resource Allocation

We now concern ourselves with the physical limitations of the receiving agent. We assume that the receiving agent has a limited resource R that it can use to receive messages. To the extent possible, the receiving resource, R, may be measured in terms of various antennas or various allocations of frequencies, etc. It is not our goal in this article to discuss the engineering of the receiving agent in general. Rather, we accept it as a given.

Upon completion of the mathematics in this section, the results do not seem surprising. That is good! It shows that our intuition is correct and it lays a foundation for dealing with many agents and non-linear allocation schemes (where we lose elements of intuition). Furthermore, aside from linearity, we based our allocation scheme on a Euclidean metric; it is not at all clear if an information geometric-style Riemannian metric be used instead. That is beyond the scope of the article.

Let us take the simplest case where there are two transmitting agent $A_{X_{1}}$ and $A_{X_{2}}$. As before, $A_{X_{i}}$ has channel matrix $M_{i}$. We model noise affecting each channel in a linear manner. Suppose that an agent $A_{X}$ is given, as before, by its channel matrix

$$M_{1}=\left(\begin{array}{cc} a & \bar{a}\\ b & \bar{b} \end{array}\right)\;.$$

How does noise, which results from the receiving agent not allocating enough of its resources to $A_{X}$, change this channel matrix? The channel (a,b) is a point in [0,1]×[0,1]. Consider the shortest path from (a,b) to the main diagonal (which consists of zero-capacity channels). View [0,1]×[0,1] as sitting $R^{2}$ and consider the straight line y=−x+(a+b). This line is orthogonal to the straight diagonal line of zero-capacity channels, goes through the point (a,b), and intersects the line for the zero-capacity channels at $\left(\frac{a+b}{2},\frac{a+b}{2}\right)$. The line segment of interest is given parametrically for t∈[0,1] as

$$\left(1-t\right)\left(a,b\right)+t\left(\frac{a+b}{2},\frac{a+b}{2}\right)\;.$$

We model noise as moving on this new line segment from the point (a,b) to the point $\left(\frac{a+b}{2},\frac{a+b}{2}\right)$. No noise corresponds to t=0, total noise to t=1; that is, we use *t* as a measure of the noise normalized in a linear manner between 0 and 1.

EXAMPLE: Let (a,b)=(0.8,0.4). If t=0, the channel is given as (0.8,0.4) and the capacity is 0.12. If t=1, the channel is given as (0.6,0.6) and the capacity is 0. Let t=0.9, then the channel is given by 0.1(0.8,0.4)+0.9(0.6,0.6)=(0.08,0.04)+(0.54,0.54)=(0.62,0.58), which has a capacity of 0.001.

Now, let t=0.1, then the channel is given by 0.9(0.8,0.4)+0.1(0.6,0.6)=(0.72,0.36)+(0.06,0.06)=(0.78,0.42), which has a capacity of .10. Note that, unsurprisingly, the cleaner channel has C(0.8,0.4)=0.1246>C(0.78,0.42).

What we have been discussing motivates the following our modeling definition.

**Definition** **3**. *An agent $A_{X}$ with channel matrix (a,b) requires the receiving resource R for its channel matrix to be unchanged. If the receiving agent only allocates A,0≤A≤R to $A_{x}$, the channel matrix is modified from (a,b) in the following manner,*

(33) $$\left(a^{A},b^{A}\right)=\frac{A}{R}\left(a,b\right)+\left(1-\frac{A}{R}\right)\left(\frac{a+b}{2},\frac{a+b}{2}\right)\;.$$

Thus, A=R corresponds to t=0 above, and A=0 corresponds to t=1 above. As A decreases, the capacity “travels” the shortest path in the Euclidean metric to the line of the 0-capacity channels. This is the essence of our modeling assumption.

Note that a channel is a 0-capacity channel iff a=b. However, if we let b=a, then $\forall A,\left(a^{A},a^{A}\right)=\left(a,a\right)$.

**Theorem** **10**. *For a non-zero channel (a,b), that is, a≠b, $C(a^{A},b^{A})$ decreases as A decreases from R to 0.*

**Proof.**  If (a,b) is a positive channel, that is, if a>b, we have that $a^{A}$ decreases and $b^{A}$ increases as A goes from R to 0. This result is easily shown with algebra, but even more simply by observation of the line segment. From ([Theorem 4.9] [6]), if (a,b) is a negative channel, then by symmetry of capacity about the line b=a, that completes the proof. □

**Corollary** **2**. *If we have a 0-capacity channel (a,b)=(e,e), then the $C(e^{A},e^{A})$ is constant at 0 as A decreases.*

**Proof.**  Trivial, since the line segment reduces to the point (e,e) is this situation. □

### 5.1. Resource Allocation Amongst Different Transmitters

Assume that there are two transmitting agents $A_{X_{1}}$ with matrix (a,b), and $A_{X_{2}}$ with matrix (c,d). The difference from before is that the receiver can only allocate total resource R to the reception by the agents and, further, each agent requires resource R to prevent degradation to its channel matrix.

If $A_{Y}$ allocates A to $A_{X_{1}}$, we have the resulting channel matrix Equation (33) as given above. Then it allocates the remainder R−A to $A_{X_{2}}$, resulting in this channel matrix

(34) $$\left(c^{R-A},d^{R-A}\right)=\left(1-\frac{A}{R}\right)\left(c,d\right)+\frac{A}{R}\left(\frac{c+d}{2},\frac{c+d}{2}\right)\;.$$

Note that

$$\begin{array}{ccc} \left(a^{R},b^{R}\right) & = & \left(a,b\right),\;\mathrm{with}\;C\left(a^{R},b^{R}\right)=C\left(a,b\right),\;\mathrm{and}\;\\ \left(a^{0},b^{0}\right) & = & \left(\frac{a+b}{2},\frac{a+b}{2}\right),\;\mathrm{with}\;C\left(a^{0},b^{0}\right)=0\;. \end{array}$$

As we have shown in the previous section, we arrive at:

(35) $$M_{2}^{1,2}{|}_{A}=\left(\begin{array}{cccc} a^{A}\cdot c^{R-A} & a^{A}\cdot \bar{c^{R-A}} & \bar{a^{A}}\cdot c^{R-A} & \bar{a^{A}}\cdot \bar{c^{R-A}}\\ b^{A}\cdot d^{R-A} & b^{A}\cdot \bar{d^{R-A}} & \bar{b^{A}}\cdot d^{R-A} & \bar{b^{A}}\cdot \bar{d^{R-A}} \end{array}\right)\;.$$

Consider the situation when all of the resource is allocated to one channel; then, without the loss of generality, we let A=R, giving

(36) $$M_{2}^{1,2}{|}_{A=R}=\left(\begin{array}{cccc} a\left(\frac{c+d}{2}\right) & a\left(1-\frac{c+d}{2}\right) & \bar{a}\left(\frac{c+d}{2}\right) & \bar{a}\left(1-\frac{c+d}{2}\right)\\ b\left(\frac{c+d}{2}\right) & b\left(1-\frac{c+d}{2}\right) & \bar{b}\left(\frac{c+d}{2}\right) & \bar{b}\left(1-\frac{c+d}{2}\right) \end{array}\right)\;.$$

Keep in mind that the above result is the channel matrix when we combine a 0-capacity channel with (a,b). Intuitively, this should not change the capacity from that of C(a,b). Looking at the channel matrix and thinking in terms of coding, we see that we are affecting the first and second outputs; as much as the third and fourth. Below, we present the mathematical details.

**Theorem** **11**. *$C\left(M_{2}^{1,2}{|}_{A=R}\right)=C\left(a,b\right)$.*

**Proof.**  Let us calculate $C\left(M_{2}^{1,2}{|}_{A}\right)$. We let $\frac{c+d}{2}:=\gamma$ and $q:=(ax+b\bar{x})$. Thus,

$$\left(y_{1},y_{2},y_{3},y_{4}\right)=\left(\gamma \left(ax+b\bar{x}\right),\bar{\gamma }\left(ax+b\bar{x}\right),\gamma \left(\bar{a}x+\bar{b}\bar{x}\right),\bar{\gamma }\left(\bar{a}x+\bar{b}\bar{x}\right)\right)\;.\;\mathrm{Then}\;\mathrm{if}\;$$

$$\left(y_{1},y_{2},y_{3},y_{4}\right)=\left(\gamma q,\bar{\gamma }q,\gamma \bar{q},\bar{\gamma }\bar{q}\right)\;,\;\mathrm{we}\;\mathrm{find}\;\mathrm{that}\;$$

H(Y)=h(γ)+h(q).

Next we examine the conditional entropy:

$$H\left(Y|X\right)=-x\left(a\gamma \log\left(a\gamma \right)+a\bar{\gamma }\log\left(a\bar{\gamma }\right)+\bar{a}\gamma \log\left(\bar{a}\gamma \right)+\bar{a}\bar{\gamma }\log\left(\bar{a}\bar{\gamma }\right)\right)\;.$$

Again use the rule that the log of a product is the sum of the logs to arrive at:

$$H\left(Y|X\right)=H\left(Y\right)-H\left(Y|X\right)=h\left(ax+b\bar{x}\right)-xh\left(a\right)-\bar{x}h\left(b\right)\;.$$

This result is the same as the mutual information of (a,b). Thus, the maximum of the mutual information for both cases remains the same. □

**Corollary** **3**. *$C\left(M_{2}^{1,2}{|}_{A=0}\right)=C\left(c,d\right)$.*

**Proof.**  If we swap the two transmitting agents we establish the proof (details are left to the reader). □

Note that any 0-capacity channel is some (a,b) channel witha 0 resource allocation. Thus,

**Corollary** **4**. *Combining (a,b) with a 0-capacity channel results in a channel with the same capacity as (a,b).*

We arrive at the question at hand—what happens with a partial allocation to each channel? That is, in general, how does $C\left(M_{2}^{1,2}{|}_{A}\right)$ compare to C(a,b) and C(c,d)? Our answer follows.

#### Allocate Resources to (a,b) and a 0-Capacity Channel

In this situation, we know that $C\left(M_{2}^{1,2}{|}_{A=R}\right)=C\left(a,b\right)$ and that $C\left(M_{2}^{1,2}{|}_{A=0}\right)=C\left(c,d\right)$. What happens for 0<A<R? Not surprisingly, we get the following theorem:

**Theorem** **12**. *Through allocation if we combine (a,b), the first channel, with (e,e), the second channel, we find that $C\left(M_{2}^{1,2}{|}_{A}\right)=C\left(a^{A},b^{A}\right)$.*

**Proof.**  Trivial from Theorem 9. □

### 5.2. More Examples

We will find the capacity of $C\left(M_{2}^{1,2}{|}_{A}\right)$ by using (35) for various A and agent matrices.

$$\begin{array}{ccc} EXAMPLE & & \;\mathrm{Given}\;a\;90/10\;\mathrm{allocation}\\ \;\mathrm{The}\;\mathrm{first}\;\mathrm{agent}\;M_{1}^{1}=\left(0.8,0.4\right), & & \;\mathrm{the}\;\sec\mathrm{ond}\;\mathrm{agent}\;M_{1}^{2}=\left(0.7,0.3\right),\;\;A=0.9\\ C(M_{1}^{1}) & = & 0.1246,\;\;C(M_{1}^{2})=0.1187\\ \left(a^{A},b^{A}\right) & = & \left(0.78,0.42\right)\\ \left(c^{R-A},c^{R-A}\right) & = & \left(0.52,0.48\right)\\ C\left(M_{2}^{1,2}{|}_{A}\right) & = & 0.1012\\ C\left(M_{2}^{1,2}{|}_{A}\right)<C\left(M_{1}^{1}\right) & & C\left(M_{2}^{1,2}{|}_{A}\right)<C\left(M_{1}^{2}\right) \end{array}$$

$$\begin{array}{ccc} EXAMPLE & & \;\mathrm{Given}\;a\;10/90\;\mathrm{allocation},\;\mathrm{with}\;\mathrm{the}\;\mathrm{same}\;\mathrm{agents}\;\mathrm{as}\;\mathrm{above}\\ \;\mathrm{The}\;\mathrm{first}\;\mathrm{agent}\;M_{1}^{1}=\left(0.8,0.4\right), & & \;\mathrm{the}\;\sec\mathrm{ond}\;\mathrm{agent}\;M_{1}^{2}=\left(0.7,0.3\right),\;\;A=0.1\\ C(M_{1}^{1}) & = & 0.1246,\;\;C(M_{1}^{2})=0.1187\\ \left(a^{A},b^{A}\right) & = & \left(0.62,0.58\right)\\ \left(c^{R-A},c^{R-A}\right) & = & \left(0.68,0.32\right)\\ C\left(M_{2}^{1,2}{|}_{A}\right) & = & 0.0967\\ C\left(M_{2}^{1,2}{|}_{A}\right)<C\left(M_{1}^{1}\right) & & C\left(M_{2}^{1,2}{|}_{A}\right)<C\left(M_{1}^{2}\right) \end{array}$$

$$\begin{array}{ccc} EXAMPLE & & \;\mathrm{Given}\;a\;90/10\;\mathrm{allocation},\;\sec\mathrm{ond}\;\mathrm{agent}\;\mathrm{has}\;\mathrm{little}\;\mathrm{noise}\\ \;\mathrm{The}\;\mathrm{first}\;\mathrm{agent}\;M_{1}^{1}=\left(0.7,0.3\right), & & \;\mathrm{the}\;\sec\mathrm{ond}\;\mathrm{agent}\;M_{1}^{2}=\left(0.99,0.01\right),\;\;A=0.9\\ C(M_{1}^{1}) & = & 0.1287,\;\;C(M_{1}^{2})=0.9192\\ \left(a^{A},b^{A}\right) & = & \left(0.6,0.4\right)\\ \left(c^{R-A},c^{R-A}\right) & = & \left(0.745,0.255\right)\\ C\left(M_{2}^{1,2}{|}_{A}\right) & = & 0.2030\\ C\left(M_{2}^{1,2}{|}_{A}\right)>C\left(M_{1}^{1}\right) & & C\left(M_{2}^{1,2}{|}_{A}\right)<C\left(M_{1}^{2}\right) \end{array}$$

From these results, we see that both

$$\begin{array}{c} C\left(M_{2}^{1,2}{|}_{A}\right)<\min\left(C\left(M_{1}^{1}\right),C\left(M_{1}^{2}\right)\right),\;\mathrm{and}\;\\ \min\left(C\left(M_{1}^{1}\right),C\left(M_{1}^{2}\right)\right)<C\left(M_{2}^{1,2}{|}_{A}\right)<\max\left(C\left(M_{1}^{1}\right),C\left(M_{1}^{2}\right)\right) \end{array}$$

are possible. In fact, equalities are also possible by using the special cases examined at the beginning of this section. However, $\max\left(C\left(M_{1}^{1}\right),C\left(M_{1}^{2}\right)\right)<C\left(M_{2}^{1,2}{|}_{A}\right)$ is not possible. (We show this by a re-wording and then proving that $\left(M_{2}^{1,2}{|}_{A}\right)$ cannot be larger than both $C(M_{1}^{1})$ and $C(M_{1}^{2})$.) Thus, we need a lemma.

**Lemma** **1**. *For channels (a,b) and (c,d), we find that*

(37) C((a,b)⊗(c,d))≤C(a,b)+C(c,d),

*with equality if a=b or c=d.*

**Proof.**  The product channel (a,b)×(c,d) is given by channel matrix

$$\left(\begin{array}{cccc} ac & a\bar{c} & \bar{a}c & \bar{a}\bar{c}\\ ad & a\bar{d} & \bar{a}d & \bar{a}\bar{d}\\ bc & b\bar{c} & \bar{b}c & \bar{b}\bar{c}\\ bd & b\bar{d} & \bar{b}d & \bar{b}\bar{d} \end{array}\right).$$

The capacity of this product channel equals the sum of the capacities of its component channels (a,b) and (c,d) (p. 85 [5]). Removing the middle two rows gives us (a,b)⊗(c,d), and, since removing a row never increases capacity, we find that

C((a,b)⊗(c,d))≤C((a,b)×(c,d))=C(a,b)+C(c,d).

□

**Theorem** **13**. *If we combine through an allocation (a,b), the first channel, with (c,d), the second channel, then $C(M_{2}^{1,2}{|}_{A})$ cannot be greater than both of the individual channel’s component capacities.*

**Proof.**  Let

$$M_{1}^{1}{|}_{A}=\left(\begin{array}{cc} a^{A} & \bar{a^{A}}\\ b^{A} & \bar{b^{A}} \end{array}\right),$$

$$M_{1}^{2}{|}_{R-A}=\left(\begin{array}{cc} c^{R-A} & \bar{c^{R-A}}\\ d^{R-A} & \bar{d^{R-A}} \end{array}\right),$$

so that $M_{2}^{1,2}{|}_{A}=M_{1}^{1}{{|}_{A}⊗M_{1}^{2}|}_{R-A}$. For any input probability distribution held constant, the mutual information is convex with respect to the elements of the channel matrix ([Theorem 2.7.4] [4]). That is, for any given input probability distribution *x*, for all $a_{1},a_{2},b_{1},b_{2},t\in \left[0,1\right]$,

$$I\left(ta_{1}+\bar{t}a_{2},tb_{1}+\bar{t}b_{2},x\right)\leq t\cdot I\left(a_{1},b_{1},x\right)+\bar{t}\cdot I\left(a_{2},b_{2},x\right),$$

where I(α,β,x) is the mutual information of channel (α,β) with input distribution *x*; thus,

$$C\left(\alpha ,\beta \right)=\max_{x}I\left(\alpha ,\beta ,x\right),\;\mathrm{and}\;$$

∴∀x,C(α,β)≥I(α,β,x).

If we let $a_{1}=a,\;b_{1}=b,\;a_{2}=b_{2}=\frac{a+b}{2},t=\frac{A}{R}$, we have from convexity that

$$\begin{array}{ccc} I(a^{A},b^{A},x) & = & I\left(\frac{A}{R}a+\left(1-\frac{A}{R}\right)\left(\frac{a+b}{2}\right),\frac{A}{R}b+\left(1-\frac{A}{R}\right)\left(\frac{a+b}{2}\right),x\right)\\ & \leq & \frac{A}{R}I\left(a,b,x\right)+\left(1-\frac{A}{R}\right)I\left(\frac{a+b}{2},\frac{a+b}{2},x\right)\left(\mathrm{this}\;\mathrm{last}\;\mathrm{term}\;\mathrm{is}\;0\right) \end{array}$$

for any input probability distribution *x*, because I(e,e,x) always equals 0. Now, we let χ be a capacity achieving input probability (unique except for 0-channels) distribution for $\left(a^{A},b^{A}\right)$, giving

$$C\left(a^{A},b^{A}\right)=I\left(a^{A},b^{A},\chi \right)\leq \frac{A}{R}I\left(a,b,\chi \right)\leq \frac{A}{R}C\left(a,b\right).$$

Therefore,

$$C\left(M_{1}^{1}{|}_{A}\right)\leq \frac{A}{R}C\left(M_{1}^{1}\right),$$

and by replacing $\frac{A}{R}$ with $1-\frac{A}{R}$ and repeating the above convexity argument, we find that

$$C\left(M_{1}^{2}{|}_{R-A}\right)\leq \frac{R-A}{R}C\left(M_{1}^{2}\right).$$

By Lemma 1,

$$C\left(M_{2}^{1,2}{|}_{A}\right)=C(M_{1}^{1}{|}_{A}⊗M_{1}^{2}{|}_{R-A})\leq C\left(M_{1}^{1}{|}_{A}\right)+C\left(M_{1}^{2}{|}_{R-A}\right)\;.\;\mathrm{Thus},$$

$$C\left(M_{2}^{1,2}{|}_{A}\right)\leq \frac{A}{R}C\left(M_{1}^{1}\right)+\frac{R-A}{R}C\left(M_{1}^{2}\right)\leq \left(\frac{A}{R}+\frac{R-A}{R}\right)\max\left(C\left(M_{1}^{1}\right),C\left(M_{1}^{2}\right)\right)\;.$$

$$\;\;\;\;\;\;\;\;\;\;\;\;\;\;\;\;\;\;\;\;\;\;\;\;\;\;\;\;\;\;\;\;\;\;\;\;\;\;\;\;\;\;\;\;\;\;\;\;\;\;\;\;\;\;\;\;\;\;\;\;\;\;\;\;\;\;\;\;\;\;\;\;\;\;\;\;\;\;\;\;\;\;\;\;\;\;\;\;\;\;\;\;\;\;\;\;\;\;\;\;\;\;\;\;\;\;\mathrm{Resulting}\;\mathrm{in},\;\;\;C\left(M_{2}^{1,2}{|}_{A}\right)\leq \max\left(C\left(M_{1}^{1}\right),C\left(M_{1}^{2}\right)\right)\;.$$

□

Thus, we have shown that $C\left(M_{2}^{1,2}{|}_{A}\right)\leq \max\left(C\left(M_{1}^{1}\right),C\left(M_{1}^{2}\right)\right)$ and, by using Theorem 11 and Corollary 3, equality can be obtained by letting A=R or 0, the choice depending on the underlying original channels.

#### Results and Discussion

In this section, we showed what happens when we have limited transmission power and want to distribute it among two transmitting agents. The theorems of this section capture the physical properties of the power allocation and happily agree with intuition.

## 6. Conclusions

We considered the use of Shannon information theory, and its various entropic terms to aid in reaching optimal decisions that should be made in a multi-agent/Team scenario. Our metric for agents passing information are classical Shannon channel capacity. Our results are the mathematical theorems in this article showing how combining agents influences the channel capacity.

We have put the idea forward of multi-agent communication on a firm information theoretic foundation. We examined simple scenarios in this paper to lay that strong foundation. We obtained results that may seem obvious, but are quite difficult to prove. We ask the reader to keep in mind that there is a big difference between “it is obvious” and “it has been shown”.

From our perspective we have shown that, except for certain boundary cases, one can achieve near perfect transmission of Shannon information, provided one has a large enough number of agents.

We have used most information versus resource (power) allocation as an optimizing criterion. With regard to resource allocation, our results tell us that the best thing to do is to just use the strongest channel. This result is not surprising. However, without the mathematics to prove it, we would be relying on intuition. Furthermore, note that we only used a simple linear allocation scheme in this section, and we only combined two agents. Future work will consider non-linear allocation schemes and multiple agents to continue what we have started in this paper. Going forward, this path is especially meaningful if we adjust the Riemannian metric to influence the power allocated to each channel. For example, a geometric region with high noise levels can be reflected in the Riemannian metric by acknowledging that the E,F,G terms of the metric are functions of *a* and *b*. We will explore this direction in future work.

In addition, in future work, we will also consider more than two agents competing for the available resources, non-Euclidean Riemannian metrics, and more complicated signaling alphabets and schemes. We are also interested in information flow in the Vicsek [19] bird flocking model.

## 7. Notation

We include some of the notation that is used repeatedly throughout the article. The other notation is variants of what we give here with changes to the indices and is made clear in its first usage.
MASMulti-agent System$A_{x}$Agent *X**M*A channel matrix, that is every row contains non-negative numbers that sum to 1$M_{n}$2 × 2*n* channel matrix, representing *n* (transmitting) AgentsH(V)Entropy of the (discrete) random variable *V*H(V|W)Conditional Entropy of the random variable *V* conditioned on *W*I(V,W)Mutual information between the random variables *V* and *W**C*Capacity of a generic channel$C_{2,2}$Specifically the capacity of a 1 (transmitting) agent channel$M_{1}^{1}$A specific 1-agent channel 
$\left(\begin{array}{cc} a & \bar{a}\\ b & \bar{b} \end{array}\right)$. Note: C(a,b):=C($M_{1}^{1}$)$M_{1}^{2}$Another 1-agent channel 
$\left(\begin{array}{cc} c & \bar{c}\\ c & \bar{d} \end{array}\right)$$M_{2}^{1,2}$The combined channel (a,b) ⊗ (c,d) with channel matrix 
$\left(\begin{array}{cccc} ac & a\bar{c} & \bar{a}c & \bar{a}\bar{c}\\ bd & b\bar{d} & \bar{b}d & \bar{b}\bar{d} \end{array}\right)$$M_{2}^{1,2}{|}_{A}$Combined power allocated channel with channel matrix
$=\left(\begin{array}{cccc} a^{A}\cdot c^{R-A} & a^{A}\cdot \bar{c^{R-A}} & \bar{a^{A}}\cdot c^{R-A} & \bar{a^{A}}\cdot \bar{c^{R-A}}\\ b^{A}\cdot d^{R-A} & b^{A}\cdot \bar{d^{R-A}} & \bar{b^{A}}\cdot d^{R-A} & \bar{b^{A}}\cdot \bar{d^{R-A}} \end{array}\right)$$M_{2-}$= $\left(\begin{array}{ccc} a^{2} & 2\bar{a}a & {\bar{a}}^{2}\\ b^{2} & 2\bar{b}b & {\bar{b}}^{2} \end{array}\right)$, formed from the (a,b) channel
